# Lactation in the human

**DOI:** 10.1093/af/vfad021

**Published:** 2023-06-14

**Authors:** Margaret C Neville

**Affiliations:** Department of Physiology and Biophysics, Department of Obstetrics and Gynecology, University of Colorado Anschutz Medical Campus, USA

**Keywords:** breast development, estrogen, human milk, progesterone, secretory activation

## Introduction

In this article, the development of the mammary gland in the human fetus is described followed by a comparison of the situation and anatomy of the mammary glands in the human with the glands in rodents and dairy animals. The hormones that bring about further development at puberty and in pregnancy are described along with the changes in the ductal structure of the gland. We consider the changes in milk composition that take place during secretory activation, the onset of milk secretion that occurs postpartum in women. The structure of milk is outlined emphasizing the various milk components obtained by differential centrifugation including whey, milk fat globules, casein micelles, extracellular vesicles, milk cells, and bacteria. Finally, we consider the minimal data addressing breast involution after weaning in the human.

## Development of the Mammary Gland in the Human Fetus

**Figure 1.**
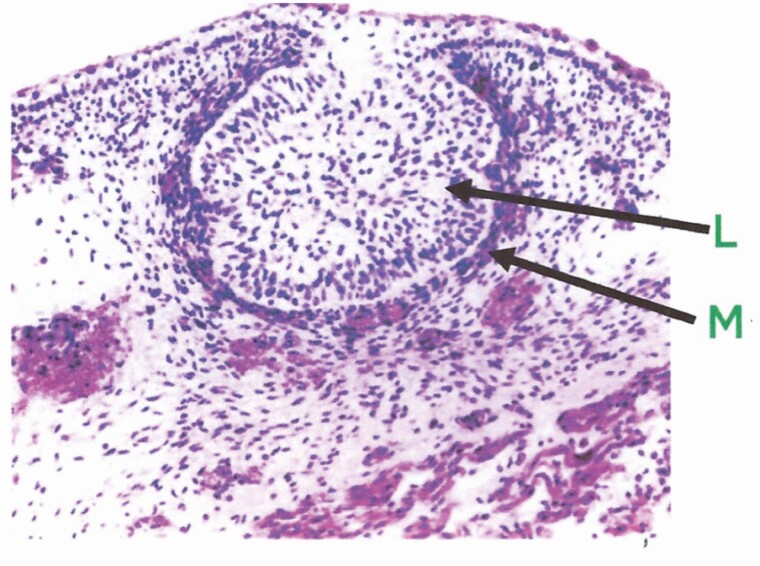
Embryonic mammary bud in a 16-wk male fetus. The epithelial cells in the lumen of the bud (L) are surrounded by a condensed layer of mesenchymal cells (M) (Howard and Gusterson, 2000). Used by permission.

In a study of fetal mammary glands of 72 human fetuses, less than 2 mo gestational age, Gusterson and colleagues (Anbazhagan et al., 1991) found the ductal system was variably developed but contained both luminal and basal epithelial cells. The fetal glands showed a chronological developmental pattern with secretory-type changes likely brought about by maternal lactogenic hormones accessing the fetus. There was evidence of milk secretion soon after birth. Involutional changes occurred by the time the infant was 2 mo of age leaving a rudimentary nipple and duct system similar in both males and females. More detailed recent studies (Gusterson and Stein, 2012) show that breast development starts about the fifth week of intrauterine life with appearance of a 2- to 4-cell-thick “milk streak” in the abdominal ectoderm extending from the chest to the groin. The thoracic area in this streak begins to thicken in the sixth or seventh week of gestation and the rest of the streak disappears. Next indentations begin to appear in this thickened region followed by the down-growth of epithelial tubes that become mammary ducts with a lumen. They appear to be lined by cuboidal epithelial cells and surrounded by mesenchymal cells (Figure 1). By the second trimester, a layer of myoepithelial cells can be discerned below the epithelial layer. In addition, adipose tissue begins to accumulate around the ductal structures. At birth, the breast tissue is similar in males and females and continues this way until puberty when hormones in the female bring about differentiation.

The molecular underpinnings of these developmental stages are known primarily from studies in mice (Hens and Wysolmerski, 2005) and include signaling by members of both the wingless-related integration site (Wnt) and fibroblast growth factor (FGF) families. In the milk streak these appear to interact with T-box family transcription factors TBX2 and TBX3. As the steak develops into placodes the members of these family appear to be joined by ectodysplasin (Eda), a member of the tumor necrosis factor ligand family. Eda is present in the mesenchymal compartment of the mouse placode and its receptor Edar is found in the epithelium. As the placode develops into the milk bud, a large number of additional proteins including insulin-like growth factor 1 (IGF-1), hedgehog (Hh), parathyroid hormone-related protein (PTHrP), neuregulin (NRG3), and their associated receptors appear to join the Wnt and FGF families in specifying developmental processes (Biswas et al., 2022). Limited information about these pathways and factors is available for the human mammary gland, which is difficult to study because of the relative unavailability of tissues.

## Developmental Changes in Puberty and Pregnancy Leading to Lactation

**Figure 2.**
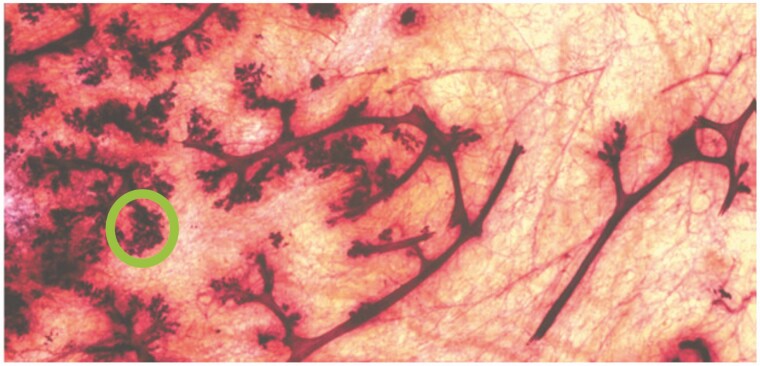
Whole mount preparation of the human breast from a 15-yr-old human female. Note the ducts with lobular structures branching from their sides and termini. Copious adipose tissue is generally present as well. A terminal duct lobular unit (TDLU) is indicated by the green circle. From Howard and Gusterson (2000). Used by permission.

Two or three years prior to the menarche, very low levels of circulating estrogen from the ovaries stimulate ductal development, acting on estrogen receptor alpha (ERα) in mammary epithelial cells. Insulin-like growth factor-1 (IGF-1), secreted by the liver or locally by stromal cells under the direction of growth hormone (GH), may foster branching of the ductal system (Macias and Hinck, 2012). As puberty progresses, small protrusions branch off the primary ducts and expand into lobular structures called terminal duct lobular units (TDLU) that become more complex as time goes by (Figure 2). These complex ductile units extend to the periphery of the gland by the end of puberty. While progesterone is added to the plasma hormones during the luteal phase of the menstrual cycle, there is conflicting evidence as to whether it stimulates TDLU growth (Lee and Kelleher, 2016). In pig mammary glands, which have significant similarity to the human mammary gland, progesterone has no effect on proliferation in the presence of estrogen and prolactin; in the absence of these hormones, it actually inhibits proliferation (Horigan et al., 2009). In the mouse, progesterone receptor knockout models show that progesterone is essential for side branching at puberty and alveolar development during pregnancy (Ismail et al., 2003; Neville et al., 2023).

In early pregnancy, secretion of estrogen and progesterone from the ovary is stimulated by chorionic gonadotropin secreted by trophoblasts of the developing placenta. By the second trimester, estrogen and placental lactogen are secreted by the syncytiotrophoblasts in the developing placenta. These hormones along with placental lactogen stimulate growth and differentiation of milk secreting structures (Lee and Kelleher, 2016). The epithelial bilayer structure is maintained throughout development including both the luminal cells, which will become the milk secreting cells, and the basal cells, which will become the myoepithelium responsible for milk ejection. The syncytiotrophoblasts in the placenta also secrete human placental lactogen, a GH-like molecule that promotes secretory differentiation probably along with prolactin (PRL) from the pituitary whose secretion increases throughout pregnancy. PRL secretion is maintained at high levels in the early period after birth. While a colostral-like secretion can be detected in the alveolar lumens, full secretion of milk is inhibited by the high concentration of progesterone present in late pregnancy (Kuhn, 1969).

The anatomy of the primate breast has been well-documented in macaques and appears to be similar to the anatomy of the human breast (Cline, 2007). It differs from that of dairy animals where ducts from the milk-secreting alveoli converge on an udder which can have 2 to 4 teats (Cunningham et al., 2005) and from rodents in which multiple ducts converge below the nipple (Cole, 1933). In mice, the ducts develop lobular structures only in pregnancy, one of the many differences between the mouse and human mammary gland. The porcine mammary gland appears to be a good model for the development of the human mammary gland (Horigan et al., 2009).

**Figure 3.**
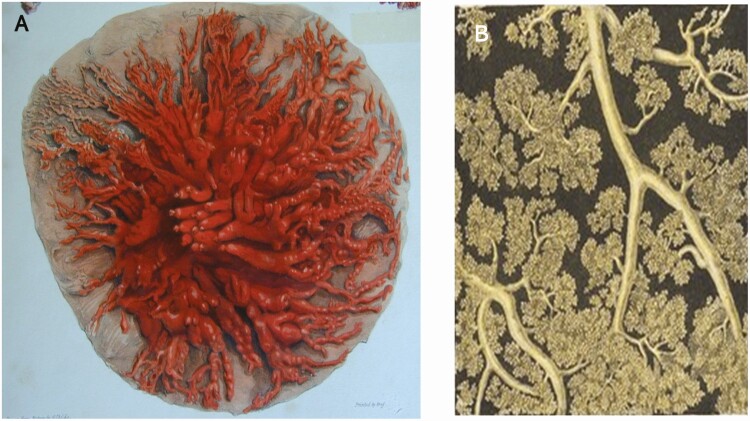
The lactating human breast. A. The ductile system of the lactating human breast. The breast of a fully lactating woman was injected with wax through the mammary ducts at the nipple by a 19th century surgeon, A.P. Cooper. B. Magnified structure of the human breast. Images by A.P. Cooper, published 1840 (Cooper, 1840).

In humans and primates, in general, multiple ductile structures terminate on the nipple as shown in Figure 3A, a famous early image of the structure of the lactating breast obtained by A.P. Copper, a surgeon in London who injected colored wax into each of the orifices terminating on the nipple (Cooper, 1840). After dissection, it could be seen that each of the ducts extends far into the breast terminating in alveoli (Figure 3B), where the milk is synthesized and held until suckling brings about oxytocin secretion. Oxytocin, in turn, causes contraction of the myoepithelial cells surrounding the alveoli and ejection of milk. Figure 3B comes from the same analysis by A.P. Cooper and magnifies the expanded alveolar structures of the lactating breast.

## Secretory Activation

**Figure 4.**
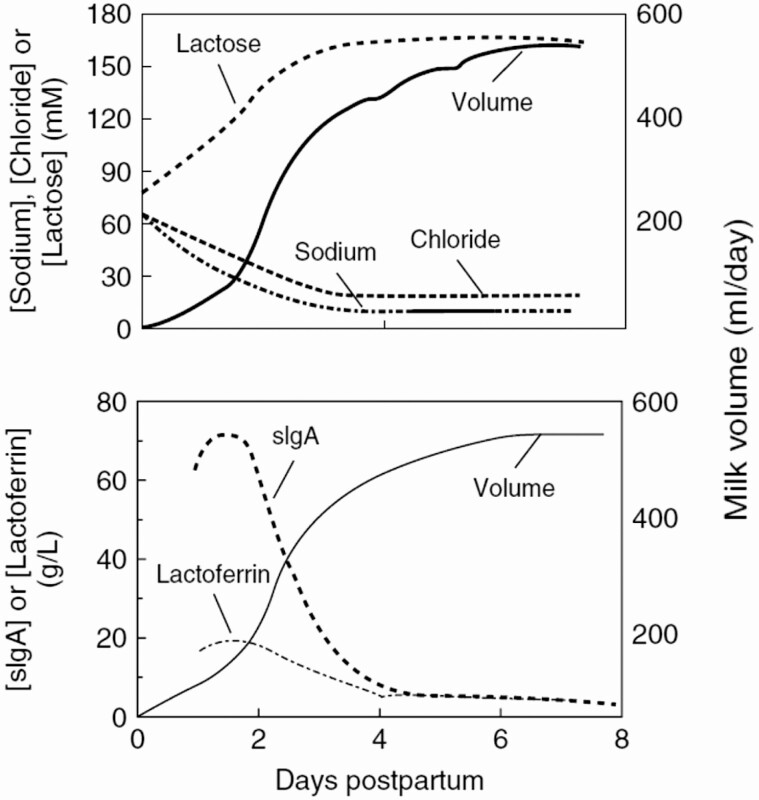
Secretory activation in women. A. Changes in milk volume, lactose, sodium, and chloride concentrations in the first 8 days after birth. B. Changes in concentrations of lactoferrin and sIgA. Inset: changes in the rate of secretion of these two milk proteins. From Neville M.C. et al. (2001), used by permission.

Milk is synthesized by the luminal alveolar cells and stored in the acini shown in Figure 3B. Milk synthesis and secretion can be altered by changes in the placental factors that govern mammary development, retained placental fragments after birth of the infant, and obesity, all of which may hinder mammary development into a fully secreting gland (Neville et al., 2023). Copious milk production is initiated in women in the first few days after birth during secretory activation brought about by a fall in progesterone and maintained prolactin (PRL; Neville et al., 2001). In dairy and other mammals, the fall in plasma progesterone occurs prior to parturition initiating lactation prior to birth. In women, however, the fall in progesterone takes place over 2 to 3 d postpartum so that small amounts of colostrum are secreted during the first few days postpartum followed by secretion of transitional milk. The first changes in composition of the breast secretion after birth are a fall in sodium and chloride and a rise in lactose concentrations reflecting closure of the tight junctions between the luminal epithelial cells (Figure 4, top). Milk volume rises more slowly representing activation of the metabolic pathways that underlie synthesis the major components of milk including casein. Concomitant with the rise in milk volume there is a fall in the concentrations of two of the major protective components of human milk, lactoferrin and secretory IgA (sIgA; Figure 4, bottom). Under normal circumstances milk volume plateaus between 500 and 600 ml/day by day 6 postpartum and increases to 700–800 ml/day by the first month, a rate that is maintained up to 6 mo of exclusive breastfeeding (Cox et al., 1996). These milk volumes appear to be characteristic of healthy breastfeeding parents throughout the world.

### Colostrum

The volume of the mammary secretion in the first 2 d after parturition (colostrum) is low, ~100 ml/day (Figure 4top). It increases rapidly up to day 4 and is classified as colostrum up to day 6. Transitional milk is often designated as milk secreted from day 10 to 14 and mature milk from day 15 on (Nyquist et al., 2022). Because humans have transplacental immunity, meaning that immunoglobulins are transmitted to the fetus across the placenta, unlike ungulate species like cattle, human colostrum is low in IgG and IgM (Bigler et al., 2022). It is, however, high in IgA which is thought to protect the gastrointestinal system from unwanted pathogens. Human colostrum also contains high levels of the protective proteins lactoferrin and lysozyme as well as larger concentrations of fat-soluble vitamins (A, E, and K) than term milk. Whether the small volume of colostrum in the first 2 d postpartum is important to the infant or whether it is mainly protective of the nonsuckled breast is a question that has not been seriously addressed. However, suckling by the infant shortly after birth results in the secretion of oxytocin, which may help parental-infant bonding by interacting with the pleasure receptors in the hypothalamus (see below; Quintana et al., 2019).

## Hormonal Regulation of Lactation

### Prolactin

**Figure 5.**
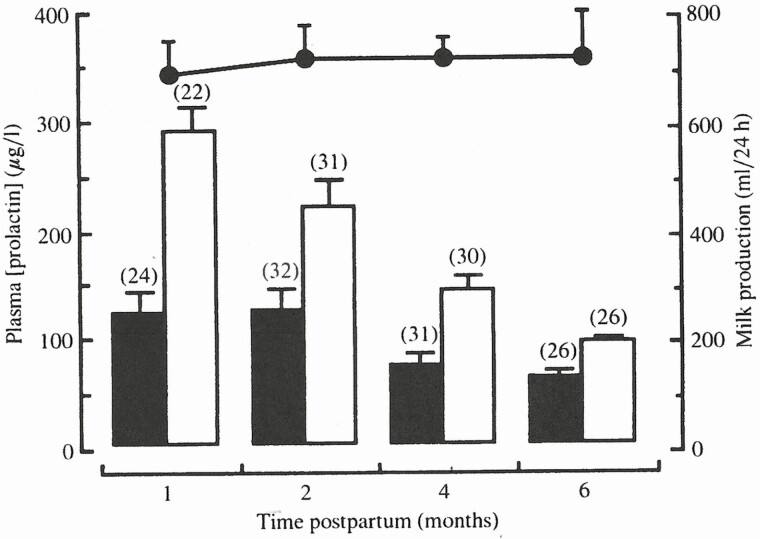
Plasma prolactin levels before (solid bars) and after (open bars) feeding as well as milk volume production (filled circles) in 11 fully breastfeeding Australian women. From Cox et al. (1996), used by permission.

High levels of PRL are present in the plasma of women at birth averaging between 100 and 300 µg/L (Figure 5). Suckling induces a significant rise in plasma PRL (Cox et al., 1996). However, plasma PRL falls with duration of breastfeeding. For example, at 6 mo in the same women basal prolactin declined about 60 µg/L rising to 90 µg/L after suckling. However, the rate of milk production did not change significantly. A predominant role of prolactin in the maintenance of human lactation has been called into question by studies showing only small effects of galactagogues such as domperidone on milk volume secretion (Grzeskowiak et al., 2019).

### Oxytocin

Suckling or other infant signals such as crying lead to oxytocin secretion by the posterior pituitary. This oxytocin travels through the blood stream to the alveoli in the breast, interacting with the myoepithelial cells whose subsequent contraction leads to milk ejection. The oxytocin also migrates to the hypothalamus where it apparently stimulates the pleasure reward centers helping bring about maternal–infant bonding (Quintana et al., 2019).

### Corticosteroids

The major corticosteroid in human plasma is cortisol. The hormone has a natural diurnal rhythm and is elevated in response to stress. Cortisol appears to be necessary to establish the functionality of human mammary cells in culture. It is thought to be necessary for secretory activation although there is data from human hair levels of cortisol indicating that stress levels of cortisol are associated with delayed secretory activation (Caparros-Gonzalez et al., 2019). The level of cortisol during lactation has been shown to be associated with changes in the lipid composition of milk (Linderborg et al., 2020; Ziomkiewicz et al., 2021).

### Insulin

This hormone has been shown to be important for mammary differentiation in the pregnant mouse (Neville et al., 2013). Its role in mammary differentiation in humans has not been elucidated. However, recent studies have shown that insulin plays an essential role in secretory differentiation, secretory activation, and mature milk production. There may also be an association between glucose intolerance and lactation problems in women.

## Properties of Human Milk

### The structure of milk

Milk is a complex ecosystem with many different components that benefit the infant in a myriad of different ways. The “whey” fraction obtained after centrifugation to remove precipitable components (see below) contains soluble proteins as well as the major carbohydrate fractions consisting of lactose (~71 g/L) and a large number of oligosaccharides (~12 g/L; Newburg and Neubauer, 1995) with diverse structures. Structural components of human milk separated by various centrifugation methods include milk fat globules, casein micelles, milk cells, exosomes, and bacteria often referred to as microbiota. We will start by discussing nutrients present in whey.

### Milk carbohydrates

Lactose and oligosaccharides are the most predominant nutrients in human milk, lactose being present at about 75 g/L compared to cow’s milk, which has only about 50 g/L (Ventrella et al., 2021). Oligosaccharides are present at about 15 g/L in mature milk and can have up to 150 different structures with about 20 structures accounting for about 90% of the total oligosaccharides (Smilowitz et al., 2023). Lactose is easily digested in the infant gut by lactase whereas oligosaccharides are poorly digested by the infant gut enzymes but are often a source of calories for the microbial population. Because oligosaccharides can attach to some microbial recognition sites on enterocytes, they also provide protection against bacterial infection. In addition, many of the milk proteins including lactoferrin, lysozyme and sIgA are heavily glycosylated with the glycosylation patterns differing over the course of lactation.

### Whey proteins

The most abundant soluble or whey proteins in human milk are alpha-lactalbumin (~3.3 g/L), lactoferrin (~2.0 g/L), sIgA (~1.0 g/L), lysozyme (29 to 96 µg/ml; Jaeser et al., 2022), bile salt stimulated lipase, and serum albumin (0.4 g/L). There are many others most of whose functions in milk are not currently clear (Smilowitz et al., 2023). *α-lactalbumin* is a component of lactose synthase, the enzyme which combines glucose and galactose to make lactose. It is the most abundant whey protein and is present in the Golgi compartment of the secreting mammary alveolar cell where it interacts with the major disaccharide synthesizing enzyme, galactosyl transferase, permitting this enzyme to utilize glucose as a substrate. *Lactoferrin*, present in high concentrations in colostrum (Figure 4), is a glycoprotein with significant antibacterial, antiviral, and even antitumor effects. It binds two ferric ions with high affinity, perhaps contributing to its bacteriostatic effects by sequestering the iron many bacteria need to function. A 49 amino acid fragment of lactoferrin, called lactoferricin, can be derived from both bovine and human lactoferrin and may have significant antibacterial activity in the infant gut as well as in the mammary gland. *Lysozyme* is another antibacterial agent present in human but not in cow’s milk. It has been shown to alter the bacterial composition of porcine milk to a more healthy microbiome (Maga et al., 2013). *Bile salt stimulated lipase* is a glycoprotein that is very effective in digesting the lipids found in milk after they enter the stomach and intestine. The *serum albumin* in human milk is derived from the plasma by transcytosis across the luminal milk secreting cell. Its concentration is about 1% of its serum concentration and its function in milk is not understood at this time. Human milk also contains a large array of growth factors, cytokines and adipokines, many of which may have functions in the infant, but are too numerous to discuss here (Smilowitz et al., 2023).

### The casein micelle

A prominent calcium and phosphate binding protein in milk is casein. Casein molecules are assembled into a structure called the casein micelle. These complex structures are 30 to 70 nm in diameter in human milk and much larger, up to 600 nm, in bovine milk. In human milk, there are two forms of casein, *β-casein* and *κ-casein* while bovine milk has another form called *α-casein* of which there are two variants α _S1_ and α _S2_. β-casein is highly phosphorylated and about half the molecular weight of human casein is due to its heavy glycosylation ~50% vs. 10% in the bovine (Lonnerdal and Atkinson, 1995). The casein micelle binds about 75% of the lysozyme in human milk (Jaeser et al., 2022); however, the activity of the bound lysozyme was not different from that of the free lysozyme.

### The milk fat globule

The lipids in milk are largely contained in spherical structures called milk fat globules; they contain a triglyceride core surrounded by a membrane made up of polar lipids and milk fat globule membrane (MFGM) proteins. The milk fat globule delivers a large variety of lipids to the infant and the lipid profile is highly variable depending on lactation stage, geographical region, and year of sampling. Lipid soluble signaling molecules like prostaglandins as well as cholesterol and sphingolipids are also present in the milk fat globule. Surface glycolipids resemble molecules that bind to specific pathogens and prevent their binding to receptors on the intestinal wall (see oligosaccharides below). The milk fat globule also contains a crescent of cytoplasm engulfed as the globule is being secreted from the alveolar cell; this is a source of mRNA that can give insight into the molecular basis of human milk secretion (Lemay et al., 2013). A recent proteomics study has revealed that human milk fat globules contain 1606 different proteins compared to macaque milk in which 518 different proteins were identified (Beck et al., 2015).

### Microbiota in human milk

Recent work has revealed that human milk contains a substantial and variable population of microbes (Smilowitz et al., 2023). The causes of the microbial variation may include diet, maternal body weight, oligosaccharide content, time postpartum, feeding practices, and many others. Milk bacteria may help to colonize the infant’s GI tract.

### Cells in human milk

It was originally thought that most cells in human milk were stem cells. However, recent detailed studies of the genome of cells specifically pelleted by centrifugation (Sharp et al., 2016; Nyquist et al., 2022) have shown that the majority of cells in healthy human milk (~82%) are lactocytes, the milk secreting cells. Several varieties of immune cells have also been identified with macrophages comprising about 50% of the immune cell population. Studies of these cell populations in various conditions associated with inadequate milk secretion have the potential to provide insight into the molecular control of milk secretion in the human. Because consistent sampling of human breast tissue has been difficult in the past, new insights into the regulation of human milk secretion are likely in the offing from advanced studies of milk cells.

### Human milk extracellular vesicles

Extracellular vesicles (hMEV) in human milk include three subpopulations: *exosomes* (40 to 150 nm in diameter), *microvesicles* (100 to 1000 nm in diameter), and *apoptotic bodies* (Chutipongtanate et al., 2022), nomenclature recommended by the International Society of Extracellular Vesicles. Formation of *exosomes* involves small vesicles which bud off the endosomal membrane into intracellular multivesicular bodies (MVB) containing many small vesicles. The MVB fuse with the apical membrane of the luminal epithelial cells allowing their internal exosomes to be released into the milk space. *Microvesicles* originate from blebbing of the apical membrane; the blebs pinch directly into the milk space. *Apoptotic bodies* (1000 to 5000 nm in diameter) are released from dying apoptotic cells. The hMEV include bodies released not only from luminal epithelial cells but possibly from the immune cells in milk. It is possible that small extracellular vesicles from cells other than milk cells may migrate across the mammary epithelium into milk. They may be floating freely in the milk or be attached to milk cells or milk fat globules. The contents of EV are derived from the cytoplasm of the secreting cells and include proteins, lipids, and microRNAs which may carry messages to the nursing infant. They have a highly variable composition which may be altered by parental physiological and pathological states.

Substantial evidence indicates that hMEV influence gut maturation and mitigate intestinal damage (Chutipongtanate et al., 2022). They can migrate intact across the infant gut epithelium and influence many developmental processes in the neonate such as the activity of immune cells and other developmental processes. They may attenuate viral infections. The composition of hMEVs is affected by maternal stress, diabetes, obesity, preterm birth, and allergic conditions possible providing a postpartum functional link between parent and child.

## Breast Involution After Lactation

Involution has been described in detail in the mouse mammary gland; because breast samples are limited in the human during involution many fewer studies exist. However, experiments showing changes in gene expression were carried out by Sharp et al. on cells harvested from human milk obtained 7 and 14 d after cessation of breast feeding (Sharp et al., 2016). The cell types present changed to resemble cells harvested from colostrum in early lactation, macrophages were increased about 10-fold, as were neutrophils and stem cells. Genes activated included STAT3, NF-kB, IRF5, and IRF7 consistent with transcription factors activated during involution in the mouse gland including Stat3, IRF’s, and NF-kB as well as p53, Smad3 (Clarkson and Watson, 2003). These findings indicate that involution is fairly rapid after cessation of milk removal in women.

## Conclusions

The biology of the human mammary gland is very different from that of rodents, which have been the source of much of our knowledge about the biology of milk secretion. Recent studies of the cells in human milk have shown that the large proportion of cells in human milk are lactocytes that retain many characteristics of milk secreting cells and offer an interesting medium for the study of the molecular biology of human lactation. Human breast milk has properties very different from most milks available commercially including milk from cows, goats, sheep, and other dairy animals. In particular, human milk is quite rich in protective proteins including lactoferrin, sIgA, and lysozyme as well as a profusion of oligosaccharides which may specifically target bacterial populations in the infant gut. 

## About the Author

**Margaret C. Neville** has been working on the functional biology of the mammary gland since the mid 70’s and is considered an international expert in this field. She published her first paper on amino acid transport in the mammary gland, in 1977 and has written over 100 peer-reviewed research papers and 80 reviews in the field. She chaired the Gordon Conference on Mammary Biology in 1999 and gave the keynote talk at that meeting in 2007. She was a founding editor of the *Journal of Mammary Gland Biology and Neoplasia,* a journal that is currently in its 27th year of publication. She was the PI on the Program Project Grant “Functional Development of the Mammary Gland” from its inception in 2000 until she turned the project over to Steven Anderson in 2011. She retired from her positions as Professor of Physiology and Biophysics and Professor of Obstetrics and Gynecology in 2013. In retirement, she continues writing and counseling activities in the fields of mammary gland and cancer biology. In 2020–2021, she chaired Working Group I of the NIH BEGIN project resulting in guidelines for research on parental factors that impact human mammary development, milk secretion, and milk composition.
